# Recent advances in the bcr-abl negative chronic myeloproliferative diseases

**DOI:** 10.1186/1479-5876-4-41

**Published:** 2006-10-11

**Authors:** Michael Bennett, David F Stroncek

**Affiliations:** 1 Department of Hematology, Ha'Emek Medical Center, Afula, Israel; 2 Department of Transfusional Medicine, National Institutes of Health, Besthesda, Maryland, USA

## Abstract

The chronic myeloproliferative disorders are clonal hematopoietic stem cell disorders of unknown etiology. In one of these (chronic myeloid leukemia), there is an associated pathognomonic chromosomal abnormality known as the Philadelphia chromosome. This leads to constitutive tyrosine kinase activity which is responsible for the disease and is used as a target for effective therapy. This review concentrates on the search in the other conditions (polycythemia vera, essential thrombocythemia and idiopathic mylofibrosis) for a similar biological marker with therapeutic potential. There is no obvious chromosomal marker in these conditions and yet evidence of clonality can be obtained in females by the use of X-inactivation patterns. *PRV-1*mRNA over expression, raised vitamin B_12 _levels and raised neutrophil alkaline phosphatase scores are evidence that cells in these conditions have received excessive signals for proliferation, maturation and reduced apoptosis. The ability of erythroid colonies to grow spontaneously without added external erythropoietin in some cases, provided a useful marker and a clue to this abnormal signaling. In the past year several important discoveries have been made which go a long way in elucidating the involved pathways. The recently discovered *JAK2 *V617F mutation which occurs in the majority of cases of polycythemia vera and in about half of the cases with the two other conditions, enables constitutive tyrosine kinase activity without the need for ligand binding to hematopoietic receptors. This mutation has become the biological marker for these conditions and has spurred the development of a specific therapy to neutralize its effects. The realization that inherited mutations in the thrombopoietin receptor (c-Mpl) can cause a phenotype of thrombocytosis such as in Mpl Baltimore (K39N) and in a Japanese family with S505A, has prompted the search for acquired mutations in this receptor in chronic myeloproliferative disease. Recently, two mutations have been found; W515L and W515K. These mutations have been evident in patients with essential thrombocythemia and idiopathic myelofibrosis but not in polycythemia vera. They presumably act by causing constitutional, activating conformational changes in the receptor. The discovery of JAK2 and Mpl mutations is leading to rapid advancements in understanding the pathophysiology and in the treatment of these diseases.

## Introduction

The concept of the myeloproliferative disorders was introduced by Dameshek more than 50 years ago [1]. He suggested then that these conditions were "closely interrelated" and due to a proliferative activity of the bone marrow by an undiscovered stimulus. In the case of one these conditions, chronic myeloid leukemia (CML), the stimulus became evident with the discovery of the Philadelphia chromosome and the translocation which results in the BCR/ABL fusion gene. This abnormal gene codes for a protein which has constitutive tyrosine kinase activity in cellular signaling pathways controlling proliferation and differentiation. The marrow is therefore chronically stimulated not due to excess external signals as suggested by Dameshek, but due to a fault in the signaling pathway itself caused by the translocation. As a result of these discoveries CML is now considered a separate entity and has a specific and effective therapy.

The other three main chronic myeloproliferative diseases are today recognized as Polycythemia Vera (PV), Essential Thrombocythemia (ET) and Myelofibrosis (IMF). These conditions are clonal disorders of hematopoietic progenitor cells which have the capacity to differentiate into excess numbers of mature cells. In PV the emphasis is on an excess production of red cells leading to a raised hematocrit and possible thrombotic complications if left untreated. Raised neutrophil and platelet counts may also be evident. In ET the emphasis is very much on an excessive production of platelets which may be markedly raised and result in a bleeding or a thrombotic tendency. The neutrophil count may also be raised but the hematocrit is normal. These two conditions are associated with a long median survival if patients do not suffer thrombotic or hemostatic complications. The third disease in this category, IMF, has however, a worse prognosis with a median survival that may be only 3.5 to 5 years [2]. In this condition there is fibrosis within the bone marrow leading to extramedullary hematopoiesis, enlargement of the spleen and bone marrow failure. Fibrosis is a secondary phenomenon due to release of cytokines from neoplastic megakaryocytes. This condition may occur in a patient with previous PV or ET, or may occur without any evident antecedent disease.

### The search for biological markers in chronic myeloproliferative disease

Molecular markers for chronic myeloproliferative diseases, such as the Philadelphia chromosome in CML, have long been searched for, but little progress has been made until recently. While visible chromosomal abnormalities can be detected in about 33% of patients with PV[3] no single abnormality is pathognomonic for PV, ET, or IMF as the Philadelphia chromosome is for CML. Recurrent chromosomal abnormalities have however been documented. A 20q deletion occurs in 10% of patients with PV and IMF, but the most common abnormality is the finding of a loss of heterozygosity of 9p in up to 33% of PV patients [4]. In ET detection of karyotype abnormalities is infrequent [3].

The lack of a chromosomal marker in PV and ET in particular leads to diagnostic difficulties. The diagnosis of both of these disorders has largely been by exclusion of conditions known to cause an elevated hematocrit or platelet count. A search has therefore been underway for many years for positive pathological features which could help in establishing the diagnosis. For many years clinical criteria was supplemented with the result of testing for clonal or abnormal hematopoiesis. More recently, molecular markers have been identified that are proving to be useful in the diagnosis of these diseases.

#### Clonal hematopoiesis

The first useful marker for chronic myeloproliferative disease was the detection of clonal hematopoiesis. Initially the use of X-inactivation patterns in females established clonality in cell lineages such as granulocytes [5-7]. However, these assays were technically demanding and not widely available.

#### Endogenous erythroid colonies

The measurement of endogenous colonies has been an important marker for diagnosing PV. In normal individuals erythroid colonies will only grow in culture media supplemented with erythropoietin (EPO). A characteristic feature of PV is the ability for erythroid progenitor cells obtained from blood or bone marrow to grow in semisolid, serum-containing cultures in the absence of EPO [8]. This "spontaneous" growth of erythroid colonies is known as endogenous erythroid colony (EEC) formation and is also observed in some cases of ET and IMF, but not in normal subjects. The formation of EEC was thought at first to be due to hypersensitivity of these cells to minute quantities of EPO in the culture media [9] as well as other growth factors such as IL-3, stem cell factor, GM-CSF, thrombopoietin (TPO) and insulin like growth factor (IGF-1) [10-13]. This augmented response of erythroid progenitors to growth factors suggested that these cells had an abnormality in the EPO signaling mechanism that controlled the processes of erythroid proliferation, maturation and apoptosis. The situation is similar in CML where the BCR/ABL protein conveys constitutive tyrosine kinase activity. Later, it became clear, however, that growth of these colonies was truly independent of EPO since it was not blocked by anti-EPO neutralizing antibodies [14].

Although the detection of EEC is a laborious procedure, requires adequate standardization and is not freely available to many centers, it is accepted as a major diagnostic criteria for PV according to WHO and European recommendations [15].

EPO levels are typically low in PV, but this finding is only accepted as a minor criteria according to these recommendations.

Spontaneous megakaryocytic colonies can also be grown without thrombopoietin in chronic myeloproliferative disease. Although first described as a useful test to discriminate between ET and reactive thrombocytosis or normal subjects [16], spontaneous colonies can also be seen in PV and IMF [17,18].

#### *PRV-1 *mRNA over-expression and CD177

The first useful molecular marker of chronic myeloproliferative diseases was the measurement of granulocyte levels of *PRV-1 *mRNA. [19] Several studies have suggested that the granulocyte over-production of *PRV-1 *mRNA was a marker of clonal hematopoieisis in PV [20-22]. These studies have found that approximately 69 to 91% of PV patients have elevated granulocyte *PRV-1 *mRNA levels. Others have found increased expression in every patient with PV [23]. This variability may be due differences in sample handling. The time that elapses prior to processing of blood samples after collection may be a crucial factor [24]. The use of whole blood rather than purified granulocytes and the choice of the housekeeping gene used to compare gene expression may also be important [25]. Granulocyte *PRV-1 *mRNA levels have also been found to be elevated in approximately 17 to 67% of patients with ET [20-22]and 46% with IMF [20].

Unfortunately elevations in granulocyte *PRV-1 *mRNA levels are not as specific for the chronic myeloproliferative diseases as initially believed. Levels of granulocyte *PRV-1 *mRNA can be transiently elevated in healthy subjects. PRV-1 is a member of the uPAR/Ly6/CD59 family of receptors. The gene *PRV-1*, and the gene encoding a neutrophil alloantigen, *NB1*, are alleles of a single gene, *CD177*, in chromosome band 19q13.31 [26]. CD177 glycoprotein can be recognized in neutrophil plasma membranes by immunofluorescence on analysis with monoclonal antibodies. In normal individuals only about 50% of neutrophils express CD177. The numbers are higher in women and increase in pregnancy, with infection and after G-CSF administration [27]. Granulocyte *PRV-1 *mRNA levels are also increased in healthy subjects given G-CSF or with infection. Neutrophil CD177 antigen density per cell is also increased in patients with PV [28], however, flow cytometry measurement of neutrophil CD177 expression cannot be used in the diagnosis of PV since the numbers of CD177 positive neutrophils is not consistently elevated compared to controls [29]. The explanation for the lack of association in PV patients between CD177 expression at the mRNA level and cell surface CD177 protein expression is not apparent. It may be due to a fault in processing or due to a release of the protein into plasma. This is the case in other similar proteins which have a GPI (glycosyl-phosphatidylinositol) attachment [30].

It appears that the increased *PRV-1 *mRNA levels are a secondary phenomenon to myeloproliferation rather than the cause of the abnormal hematopoeisis. Other useful biomarkers such as high plasma vitamin B12 levels and raised neutrophil alkaline phosphatase scores are also secondary events

### JAK2 V617F

A specific mutation in the Janus kinase, JAK2, has been identified in the majority of patients with PV and in many patients with ET and IMF. The presence of this somatic mutation in myeloid cells has proven to be more specific for myeloproliferative diseases than elevated PRV-1 mRNA levels and its assessment has now become a standard molecular assay for the diagnosis of PV.

#### The Janus Kinases

The receptors for EPO, G-CSF, GM-CSF, TPO and some interleukins are termed type I cytokine receptors. These are characterized by a lack of a cytoplasmic tyrosine kinase domain and instead use an intracellular JAK-STAT pathway to initiate signaling [31]. The STAT pathway refers to **S**ignal **T**ransducers and **A**ctivators of **T**ranscription which are responsible for directly influencing gene expression. JAK refers to the **Ja**nus **K**inases, named after the Greek-Roman god of gates and passageways. Initially one kinase involved in this system was identified and this was skeptically nicknamed "**j**ust **a**nother **k**inase". Three more members of this family have been found and they are now recognized to be crucially important in growth factor and cytokine signaling. JAK-2 was the third Janus kinase discovered and it is involved in haemopoietic receptor signaling for EPO, G-CSF, GM-CSF and TPO.

JAKs bind to juxtamembrane cytoplasmic regions of type I cytokine receptors by their FERM (**F**our-point-one, **E**zrin, **R**adixin, **M**oesin) domain. In the unliganded state, the receptor exists as a dimer separated by 73 Å. After binding with an activating ligand, a conformational change occurs resulting in the reduction of the separation of the two strands to 39 Å [32]. This allows close apposition of two bound JAK-2 molecules and their mutual activation by cross – phosphorylation. Once activated JAK-2 exerts kinase action via its JH1 domain initially on the receptor itself providing docking sites for STATs and later for other signaling and regulatory elements.

**Figure 1:**
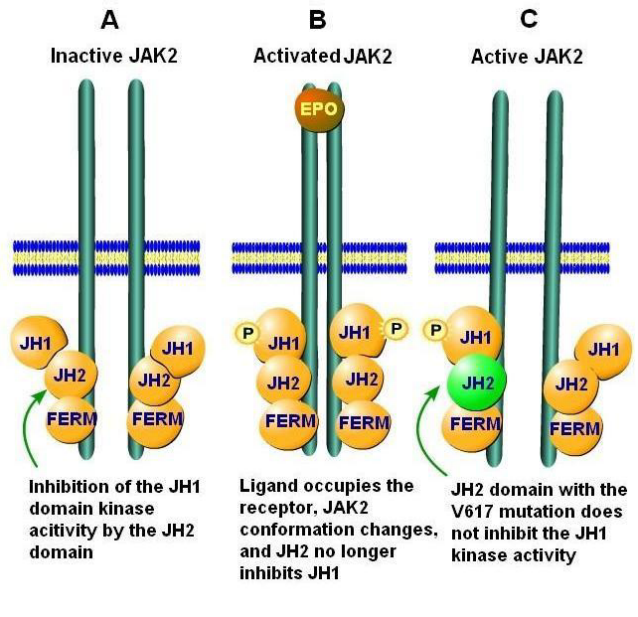
**The domains of JAK2 illustrating binding to the receptor and changes consequent to receptor binding and mutation in the JH2 domain**. The V617F mutation of the JH2 domain of JAK2 results in constitutive kinase activation. Panel A: When no ligand in bound to the EPO, TPO, G-CSF or GM-CSF receptors, the kinase activity of the JH1 domain is inhibited by the JH2 domain and JAK2 is inactive. Panel B: When EPO binding to it receptor, the two strands of the receptor come closer together, JAK2 changes conformation, the JH1 kinase activity in no longer inhibited by JH2. Panel C: The *JAK2 *V617F mutation prevents JH2 from inhibiting JH1 and the kinase is active even when no ligand is bound by the receptor.

In addition to the FERM domain, JAK2 has two other domains JH1 and JH2. The JH1 domain is situated near the carboxyl terminal of the protein. Proximal to this is a JH2 domain which has no kinase activity, but normally prevents the kinase activity of JH1 by interacting with its activation loop. When ligand binds, the resulting conformational changes in the receptor and in the bound JAK-2 cause a separation of the JH2 inhibitory domain from JH1. This leads to the expression of kinase activity (Figure 1) [33,34].

#### The importance of the *JAK2 *V617F mutation

The critical role of JAK2 in signal transduction by hematopoietic growth factor and cytokine receptors suggested that JAK2 might have abnormal tyrosine kinase activity in chronic myeloproliferative disease. In Drosophila a mutation in the JH2 domain causes a leukemia like picture due to hyper activation of the JAK-STAT pathways [35]. In the spring of 2005, almost simultaneously, five reports appeared describing a mutation in the gene coding for JAK2 also in this pseudo kinase JH2 domain, in patients with chronic myeloproliferative diseases [36-40]. This was a G to T mutation at nucleotide 1849, which leads to phenylalanine being substituted for valine at codon 617 (V617F). This mutation leads to a lack of inhibition of the JH1 domain and constitutive JAK2 kinase activity without the coupling of ligands to hemopoietic receptors.

The mutation occurs mainly in PV with a frequency of between 76% and 97% depending upon the method of detection used and the accuracy in the diagnosis of PV. It is less common in patients with ET (29–57%) and IMF (50%). Most patients have a heterozygous mutation, but in 30% the mutation is homozygous due to mitotic recombination corresponding to LOH and more advanced disease [4,38,39,41]. Analysis of BFU-e cells after cell culture however, has shown homozygous mutations in all cases of PV, but not in ET unless they developed a PV phenotype [42]. This mutation is found only occasionally in other hematological diseases: 3% in patients with chronic myelomonocytic leukemia, 5% in other myelodysplastic syndromes, 2 out of 8 patients with systemic mastocytosis, 1 out of 6 with chronic neutrophilic leukemia and none out of 11 patients with the hypereosinophilic syndrome [69]. It has also been found in 6 out of 9 patients with refractory anemia with ringed sideroblasts and thrombocytosis [43].

*JAK2 *V617 very likely has an important role in the pathobiology of PV, since it gives cells with the mutation a proliferative advantage. In vitro studies have found that cells transfected with *JAK2 *V617 had a proliferation and survival advantage over wild type *JAK2 *transfected cells. The *JAK2 *V617F transfected cell were more sensitive to stimulatory signals [44]. Cells transfected with *JAK2 *V617F were also able to activate STAT mediated transcription in the absence of erythropoietin, but cells transfected with wild-type *JAK2 *did not [37]. Mice transplanted with bone marrow cells infected with murine *JAK2 *V617F developed erythrocytosis four weeks after transplant but not mice transplanted with cells transfected with murine wild-type *JAK2 *[37].

In ET, patients with *JAK2 *V617F have a significantly higher Hb level than without the mutation [45-47], suggesting that these patients are more similar to PV. The detection of this mutation by PCR in ET which can detect 2–4% of mutated cells, is more sensitive as a test for clonality than X-chromosome inactivation patterns which require at least 26% mutated cells. There are, however, cases of ET who have monoclonal hematopoiesis according to X-chromosome inactivation patterns but are negative for the JAK2 mutation [48], suggesting that in these another mutation may be present. This may also be the case in those patients with true PV who have EEC and monoclonal hematopioesis but who do not have the *JAK2 *mutation.

Many of the markers previously useful in the diagnosis of PV and ET may be due to a downstream effect of JAK2 activation. The myeloproliferation that results from constitutive JAK2 activation could result in increased neutrophil alkaline phosphatase scores, increased levels of vitamin B_12 _(due to increased transcobalamin I) and *CD177 *mRNA overexpression. Interestingly, *CD177 *mRNA is also overexpressed by neutrophils from healthy subjects given G-CSF [28]. Since G-CSF stimulates neutrophils by binding to the G-CSF receptor and activating JAK2, this provides further evidence that JAK2 overactivation is likely responsible for the overexpression of *CD177 *mRNA in patients with PV.

The fact that JAK2 constitutive activation due to the V617F mutation occurs in PV, ET and IMF suggests that those with this mutation are part of a common disease with different expression due to other factors such as disease duration. It is also possible that the different phenotypes are due to additional mutations influencing for instance megakaryocytes and platelet production. This has now become apparent with the discovery of c-Mpl mutations in some cases (see later).

Using very sensitive techniques the *JAK2 *V617F has been detected in up to 10% of healthy donors with normal blood counts [49]. This suggests that the mutation may occur as an early event in the development of the disease or other factors are required for disease development. This is seen in CML where BCR/ABL expression can occur in normal individuals using sensitive techniques [50,51]. BCR/ABL specific T cells have also been found in healthy individuals [52] which suggests an immune mechanism for control of the disease.

### Mutations in Cytokine receptors

The possibility that an acquired mutation in a cytokine receptor may be responsible for myeloproliferative disease has been suspected but until now none have been found. Inherited mutations with a proliferative phenotype are however occasionally seen. With the erythropoietin receptor (EpoR) for example, a truncated receptor with loss of the cytoplasmic carboxyl terminal leads to familial polycythemia [14]. In this mutation there is loss of the docking site for SHP1 which normally would dephosphorylate JAK2. Prolonged phosphorylation of JAK2/STAT5 and prolonged erythropoietin "on" signals lead to a high hematocrit.

Mutations have been found in the G-CSF (G-CSFR) receptor in some patients with severe congenital neutropenia [53]. These initially have germline ELA-2 mutations which cause neutrophil elastase deficiency and then develop a secondary somatic mutation in the G-CSFR. This occurs in the terminal cytoplasmic region responsible for the binding of SHP-1 and SOCS (suppression of cytokine signaling) proteins leading to a hyperproliferative response [54]. Many of these patients go on to develop acute myeloid leukemia.

### The thrombopoietin receptor (c-Mpl)

Abnormalities of the thrombopoietin receptor known as c-Mpl have been suspected of being involved in myeloproliferative diseases. In fact, c-Mpl was initially identified as a proto-oncogene when its transmembranous and intracellular domains were transduced into the envelope of the myeloproliferative leukemia virus (MPLV) [55]. In mice this virus causes a generalized myeloproliferative polycythemic-like disorder with granulocytosis, thrombocytosis and erythroblastosis [56]. This proliferation might be due to loss of the extracellular domain which is thought to exert an inhibitory effect. Truncation of this area of c-Mpl causes TPO independent growth [57]. TPO might exert its influence by leading to a relief of this block.

The expression of c-Mpl has been found to be reduced in ET [58], PV and IMF [59]. This is related to incomplete c-Mpl glycosylation rather than c-Mpl gene disruption or transcriptional repression [60]. The expression of c-Mpl is, however, also reduced in reactive thrombocytosis [61] and in hereditary thrombocytosis caused by a TPO gene mutation [62], showing that these findings are not specific for myeloproliferative disease.

### Inherited mutations in c-Mpl

**Figure 2:**
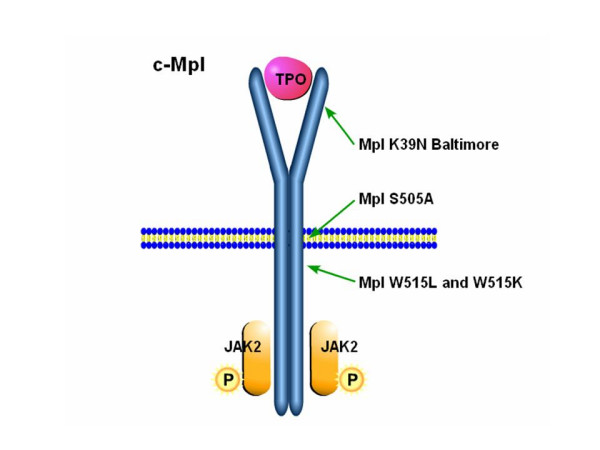
**Polymorphism and mutations of the thrombopoietin receptor, c-Mpl**. Two polypmorphisms, c-Mpl K39N and S505A, lead to constitutive activity of c-Mpl and thrombocytosis. Somatic mutations in the juxtamembranal domain of c-Mpl also lead to constitutive activity and have been found in patients with ET and IMF.

The recent discovery of two inherited mutations in *c-Mpl *has fueled the search for acquired mutations in these disorders. The first inherited mutation discovered was in a Japanese family known to have "Familial" Essential Thrombocythemia inherited in an autosomal dominant manner. Here there is an amino acid change from serine to asparagine in position 505 in the receptor (S505A), resulting from a heterozygous G to A nucleotide substitution at position 1073 in exon 10 of *c-MPL *[63]. This mutation occurs in the transmembranous portion of the receptor and leads to constitutive activity of the receptor. The second c-Mpl mutation found was Mpl Baltimore, K39N [64], which is a polymorphism near the terminal portion of the extra-cellular portion of the receptor (Figure 2). This results from a single base change guanosine to thymidine at nucleotide 1238 in exon 2 and is found exclusively in African Americans with a gene frequency of 7%. Heterozygotes have elevated platelet counts as compared to controls and homozygotes have even higher counts, in excess of 800 × 10^9^/L.

The reason why these *c-Mpl *polymorphisms should cause thrombocytosis is not entirely clear. They could allow the receptor to assume an active configuration involving the closer proximity of the two strands of the receptor dimer and consequent JAK2 activation.

### Acquired 515 mutations in c-Mpl

Recently it was suggested that abnormalities within a 5-amino acid amphipathic motif (RWQFP in humans) which is a juxtamembranal domain of the c-Mpl receptor, leads to constitutive activation [65]. This area, which extends from 514 to 518, is thought to prevent the two strands of the c-Mpl dimer from approximating when ligand is not bound to the receptor (Figure 2). Two acquired mutations have now been found at position 515 within this motif, W515L [66] and W515K [67]. These reports present the first evidence of a somatic mutation in a cytokine receptor in chronic myeloproliferative diseases. In 1182 patients '515' mutations were found in 20 [67]. Amongst the ET patients mutations were found in 1% and amongst the IMF patients they were found in 4–5%. The mutation was seen in 6 patients who were also *JAK2 *V617F heterozygous. It was not clear whether the JAK2 and c-Mpl mutations were in two separate clones or represent two mutations in the same clone. Two cases had a mixed clonal state with both '515' mutations. The 515 mutations were not seen in PV suggesting in this disease *JAK2 *V617F is prominent and is related to the phenotype of red cell production. In contrast, c-Mpl mutations affect megakaryocytes and lead to IMF and ET. The MPL515 mutations that have been detected thus far described are infrequent, but it is expected that further c-Mpl mutations will be discovered.

## Conclusion

The identification of specific molecular mutations in patients with chronic myeloproliferative disease has been a significant clinical advancement. There is no doubt, that the discovery of the *JAK2 *V617F mutation represents a major advance in understanding the pathophysiology of these disorders. It is an aid to diagnosis in some cases and will no doubt lead to the development of specific therapy in the future.

The identification of granulocyte *JAK2 *V617F is highly specific for the diagnosis of PV and has rapidly replaced other biochemical, cellular, and molecular diagnostic assays. The presence of *JAK2 *V617F is also useful in evaluating patients with elevated platelet counts who are suspected of having ET, although only approximately half of all ET patients have *JAK2*V617F. Many clinical investigators are evaluating c-Mpl for mutations and polymorphisms in these ET patients with and without *JAK2 *mutations. Some ET patients will have c-Mpl mutations, but the exact role of c-Mpl mutations in ET is not yet certain. ET is certainly a heterogeneous disease. It needs to be separated from IMF and prodromal IMF by careful histological analysis of bone marrow biopsy specimens [68] and from latent PV. The importance of the polyclonal versus the monoclonal forms of the disease need to be ascertained. It is hoped that molecular markers will help in this regard.

The identification of an activating mutation in JAK2 as a cause of chronic myeloproliferative disease could have important clinical implications. The development of specific inhibitors of the BCR/ABL kinase found in patients with CML has had a remarkable clinical impact. They are highly effective and have little toxicity. Specific inhibitors of the abnormal JAK2 kinase are not yet available, but will likely be tested and available in the near future. Since the therapeutic modalities available to treat chronic myeloproliferative diseases are limited to phlebotomy, platelet inhibitors, cytotoxic drugs and in some cases allogeneic stem cell transplantation, the availability of an effective and specific molecular therapy would be an enormous advance.

While mutations in JAK2 may not be the molecular defect that initiates PV, ET or IMF, the discovery of this molecular marker has made the diagnosis of PV and ET simpler, faster and more precise. The more precise diagnosis and classification of these patients will surely lead to more rapid advances of clinical investigations aimed at further defining the pathophysiology and more effective treatment of these diseases.

Dameshek showed outstanding foresight in recognizing and grouping together the chronic myeloproliferative diseases. This has lead to developments in our understanding of their common pathophysiological mechanisms and the peculiar molecular events in each disease category resulting in different phenotypes. These are exciting times with new discoveries coming at a tremendous pace. All of this will no doubt eventually lead to the development of specific therapy and improved care for these patients.

## Competing interests

Both authors were involved in composing this manuscript and both declare that they have no competing interests in the publication of this article.
